# Factors Associated With Preference of Psychological Intervention and Mental Status Among Chinese Teachers During Coronavirus Disease 2019: A Large Cross-Sectional Survey

**DOI:** 10.3389/fpsyt.2021.704010

**Published:** 2021-07-19

**Authors:** Xu Lizhi, Cheng Peng, Zheng Wanhong, Xu Shengmei, Li Lingjiang, Zhang Li, Wang Xiaoping, Li Weihui

**Affiliations:** ^1^Department of Psychiatry, Second Xiangya Hospital, Central South University, Changsha, China; ^2^Mental Health Institute, Second Xiangya Hospital, Central South University, Changsha, China; ^3^Key Laboratory of Psychiatry and Mental Health of Hunan, Central South University, Changsha, China; ^4^Department of Behavioral Medicine and Psychiatry, West Virginia University, Morgantown, WV, United States; ^5^University of Illinois, Urbana-Champaign, Champaign, IL, United States

**Keywords:** teachers, mental health, COVID-19, psychological intervention, anxiety, sleep disturbance

## Abstract

**Aims:** The authors sought to explore the psychological distress of teachers during COVID-19 pandemic and their preference for psychological intervention. The overarching goal was to gain insight on how to build an effective psychological support system for teachers during and after the pandemic.

**Methods:** The mental health condition of teachers (*N* = 18,521) was assessed online by using a questionnaire consisting of standard instruments PHQ-15, GAD-7, PHQ-2, PC-PTSD, and additional questions about sleep disturbance, suicidality and preference of psychological intervention methods.

**Results:** 35.5% of Chinese teachers reported sleep disturbance, 25.3% complained somatic discomfort, 17.7% had anxiety symptoms, 4.0% had depression, 2.8% had self-injury or suicidal thoughts. Women are more likely to have somatic symptoms, sleep disturbance and depression. There were age differences for anxiety, somatic symptoms and suicidal thoughts. High percentages of university teachers reported moderate to severe anxiety, somatic symptoms, depression and sleep disturbance. The most preferred psychological intervention is the self-practice of stress management skills (*N* = 11,477, 62.0%). Teachers with moderate and severe symptoms are more likely in need of hotline and online counseling and those with serious suicidal thoughts are three times more likely to use a telephone hotline.

**Conclusions:** During the COVID-19 outbreak, the major reported psychological distresses among Chinese teachers are anxiety, sleep disturbance and somatic symptoms. There were gender, age and school setting differences. Females, teachers over 45 years old and those who work at universities tend to be more vulnerable. Different teachers chose different interventions, mostly based on the severity of their symptoms.

## Introduction

Many studies have demonstrated a clear linkage between major infectious disease outbreak and its impact on mental health. For example, the outbreak of Severe Acute Respiratory Syndrome (SARS) in 2003 led to significantly increased number of mental illness cases and prolonged courses. A survey found that 38.9% of general population were worried about health problems caused by SARS, among which women and people with low education reported higher level of anxiety (1).

The COVID-19 has caused a worldwide pandemic that affected every aspect of human lives. The new pathogen was found to be more infectious than SARS-CoV (2). Many countries have started different measures to mitigate the transmission of the virus. Most public services including schools have to be closed and people are encouraged or required to do social distancing and home isolation. While stringent measures to keep people apart can slow the spread of the virus, they may come with significant mental health cost. One study during the SARS period found that among faculty and students quarantined in Beijing, 24.6% met diagnostic criteria for psychiatric disorders during the quarantine, and 26.2% had problems 8 months after the quarantine ended (3). Of the 129 citizens voluntarily quarantined in Toronto, 28.9% experienced symptoms of post-traumatic stress disorder (PTSD) and 31.2% had depression.

Teaching is a stressful and challenging profession. During this pandemic time, in addition to isolation, teachers have to adapt to many other changes that could potentially make them more vulnerable to psychological distress. The impact of COVID-19 and school closure on the mental health of teachers is unclear but warrants research. This is not only because of the importance of teachers in our society but also due to the comparative influence of teachers on students and parents. Researches show that teachers' stress and negative emotions can lead to poor classroom performance (4) and affect their ability to properly support and respond to students (5). The so-called teacher-oriented teaching model, that is, teachers play the role of classroom lecturers, presenting information directly to students, and the subsequent high pressure on Chinese teachers make this population unique from those of other countries. The present study aims to understand the psychological distress of Chinese teachers during COVID-19 pandemic and their preference for psychological intervention. The overarching goal was to gain insight on how to build an effective psychological support system for teachers during and after the pandemic.

## Materials and Methods

The data of this study were obtained through an online questionnaire. A hyperlink was distributed via WeChat social media platform and emails. The completion of the survey was voluntary and anonymous. After submission, the participants were given a choice to download electronic copies of some psychology educational materials, audio instructions for stress management, as well as a list of professional hotline and online counseling services. The study was approved by the Ethics Committee of the Second Xiangya Hospital of Central South University.

### Participants

The survey was online distributed to teachers of kindergartens, primary schools, middle/high schools and universities in Changsha on February 21 and officially closed on February 29, 2020. 18,521 teachers fully read and signed the online informed consent form before filling out the questionnaire, and voluntarily participates in the survey. As the survey was posted online, only those who completed the questionnaire and clicked the submission were counted. We were not able to collect data on incomplete responses. The survey was conducted in Changsha, Hunan Province because the number of schools and universities in Changsha allowed a huge sample size and the strong support from the Changsha Municipal Bureau of Education provided convenience for the survey distribution and data collection.

### Questionnaire Measures

The questionnaire of this study is a combination of four standard self-administered instruments and some customized specific questions.

We used PHQ-2 for assessment of depression. The cut-off score for significant clinical symptoms is 3 (6). GAD-7 was used to screen anxiety. Cutoff points 5, 10 and 15 represent mild, moderate and severe level of anxiety symptoms (7, 8). PHQ-15 was included for measuring the severity of somatic symptoms (9). The cutoff scores of 5, 10 and 15 are used for mild, moderate and severe level of somatization (6). For PTSD, we used PC-PTSD-5. A cut-off score of 4 is used in this study because of the reported well-balanced specificity and sensitivity therefore maximal efficiency (10).

We added two items to screen for sleep, self-injury and suicidal ideation. Item 9 of PHQ-9 was used to screen self-injury and suicidal ideation in the past 2 weeks. Another separate sleep item also from PHQ-9 asks subjects having difficulty falling asleep or restless or sleeping too much in the past 2 weeks. This item can be used for a wide range of sleep screening, and its performance is comparable to Insomnia Severity Index (ISI) (11). Last, we included a question about preference for psychological intervention. Four choices were given: self-care and self-reading of psychology materials, self-adjustment (stress management skills), telephone hotline, and online psychological counseling. The reason for choosing these interventions is because at the beginning of the epidemic, our mental health center has compiled a list of professional e-books, set up a telephone hotline and launched an online consultation platform. Those had become easily accessible to the public at the time of this survey. Also the previous experiences of using telephone hotline and online services in major disasters support the usability and applicability of these two approaches in the current pandemic (12, 13).

### Statistical Analysis

Descriptive statistics were used to explore the demographic characteristics of the participants, the frequency, concentration trend of psychological symptoms. The severity of symptoms was distinguished according to the scores of different scales. The detection rates were counted by percentages. Chi-square tests were used to analyze the possible relationship between the incidence of different degrees of symptoms and the characteristics of patients. A *Post hoc* analysis is performed when the chi-square test finds a statistical significance. We calculated the Adjusted Standardized Residuals (ASR) to find out which cells in the contingency table are different from their expected values. The larger the ASR, the greater the contribution of these residuals to the overall chi-square test. To estimate more conservatively, we chose to limit the absolute value of the ASR to 3 (42). When it is over 3, we think that there is a statistically significant difference between the observed and the expected frequency.

## Results

### Demographic Characteristics

A total of 18,521 people responded to the survey. Table 1 summarizes the demographic characteristics. 78.9% of participants are women, which is consistent with the gender distribution of teaching profession in China. Over 90% are elementary, middle/high school teachers, which are the main targeted population of this study. Among the four age groups, 26 to 35 years old accounts for the highest proportion (39.2%). For educational background, most have undergraduate degrees (76.6%). While 93.1% teachers were living with family at home, 5.7% reported home isolation from other family members, a few were active volunteers in COVID-19 taskforce, 6 had confirmed COVID-19 infection, 1 had suspected infection. Also, 763 (4.1%) participants reported flu-like symptoms or other general physical discomfort that were not caused by COVID-19. Of all subjects, only 122 (0.07%) reported known exposure to COVID-19 infection.

**Table 1:** Demographic characteristics (*N* = 18,521).

**Sex *N* (%)**
Male	3,909 (21.1%)
Female	14,612 (78.9%)
**School Setting** ***N*** **(%)**
Kindergarten	1,436 (7.8%)
Primary School	9,764 (52.7%)
Middle/High School	6,994 (37.8%)
University	327 (1.8%)
Age *N* (%)
≤ 25	3,023 (16.3%)
26–35	7,269 (39.2%)
36–45	4,364 (23.6%)
>45	3,865 (20.9%)
**Education** ***N*** **(%)**
Associate Degree	2,937 (15.9)
Bachelor's Degree	14,178 (76.6)
Master's Degree	1,342 (7.2)
Doctor's Degree	64 (0.3%)
**Surrounding risk of infection** ***N*** **(%)**
Staying at home with family	17,246 (93.1%)
Home Isolation from family	1,062 (5.7%)
Active volunteering	90 (0.5%)
Confirmed infection, in treatment	6 (<0.1%)
Suspected infection, in quarantine	1 (<0.1%)

### Prevalence Rates of Anxiety, Depression, Somatic Symptoms, PTSD, Suicidal Thoughts, and Sleep Disturbance

**Figure 1:**
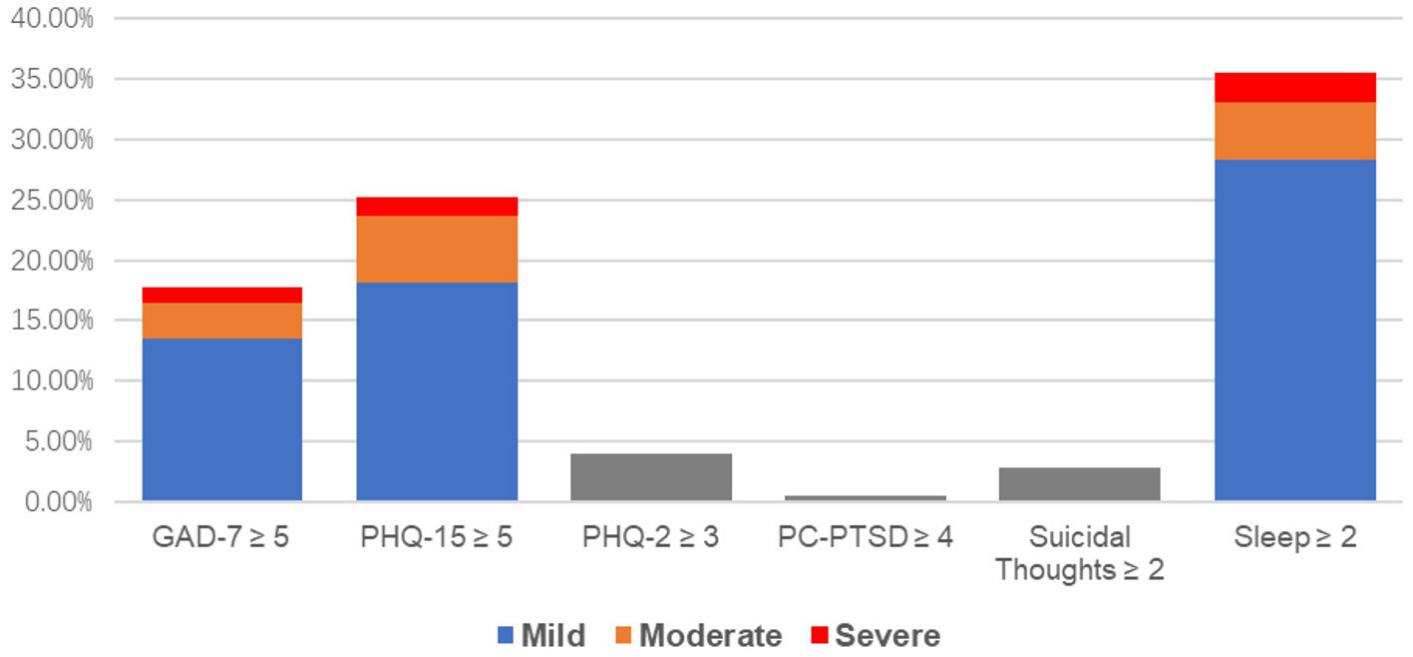
Prevalence rates for symptomatic ratings. 1. For GAD-7 and PHQ-15 the cutting score is 5. 2. For PHQ-2 the cutting score is 3. 3. For PC-PTSD the cutting score is 4. 4. For Suicidal thoughts and Sleep, symptomatic means at least “more than half of the days” in the past 2 weeks.

Figure 1 shows 17.7% of teachers scored ≥ 5 (at least mild anxiety) on GAD-7, of which 4.2% reported moderate to severe anxiety (GAD-7 score ≥ 10). For depression, 4.0% had PHQ-2 score ≥ 3, interpreted as having significant clinical depression. 25.3% teachers scored ≥ 5 on PHQ-15 for somatic discomfort, among which 5.5% had moderate and 1.5% had severe symptoms. Sleep, general fatigue and back pain were the three most frequently reported somatic discomforts. The high level of sleep complaint was self-validated by a separate sleep item, for which 35.5% reported sleep disturbance for a few days to almost every day. Surprisingly, only 0.5% teachers scored ≥ 4 on PC-PTSD scale. The number of people with suicidal thoughts or self-injury accounted for 2.8% of the total population. Among these, 0.3% reported having suicidal thoughts more than half of the days in the past 2 weeks and 0.2% almost every day.

### Correlation Analysis

Table 2 summaries the correlation analysis between demographic characteristics and scores of GAD-7, PHQ-15, and PHQ-2. We divided the subjects into two groups: no to mild symptom group, and moderate to severe symptom group. For anxiety, there were significant differences between these two groups in terms of age (X^2^ = 93.72, *P* < 0.001) and school section (X^2^ = 38.83, *P* < 0.001). Teachers over 45 years old (ASR = 7.7) and those who work in universities (ASR = 4.7) were more likely to have moderate to severe anxiety symptoms. While gender and education did not seem to have an association with the severity of anxiety symptoms, it is not true for somatic complaints. 7.6% of the female teachers reported moderate to severe somatic discomfort, which was statistically higher than their male counterparts (4.9%). The higher education, the more likely the teacher will report somatic discomfort. The same trend was noticed in age and school section where teachers work. 8.5% of the teachers over 45 years old (ASR = 4.0) and 12.8% of university teachers (ASR = 4.1) reported moderate to severe somatic symptoms. To our surprise, none of the six patients with confirmed infection reported severe physical discomfort (data not shown). For depression, more females had moderate to severe symptoms (X^2^= 8.447, *P* < 0.01). Again, higher percentage (8.6%) of university teachers endorsed moderate to severe depression. Chi-square showed no statistical difference in depression severity in terms of age and education background.

**Table 2:** Demographic and GAD-7, PHQ-15, PHQ-2, Chi-square analysis.

	Anxiety symptoms^*^		**χ^2^ (df)**	Somatic Symptoms^**^		**χ^2^ (df)**	Depressive symptoms^***^		**χ^2^ (df)**
**Demographic Characteristics**	**Non to mild *N =* 17,735**	**Moderate to severe *N =* 786**		**Non to mild *N =* 17,218**	**Moderate to severe *N =* 1,303**		**Asymptomatic *N =* 17,784**	**Symptomatic *N =* 737**	
**Sex**			1.54 (1)			34.16 (1) ^§^			8.45 (1)^‡^
Men, *N* (%)	3,757 (96.1)	152 (3.9)		3,717 (95.1)	192 (4.9)		3,785 (96.8)	124 (3.2)	
ASR^****^	1.2	−1.2		5.8	−5.8		2.9	−2.9	
Women, *N* (%)	13,978 (95.7)	634 (4.3)		13,501 (92.4)	1,111 (7.6)		13,999 (95.8)	613 (4.2)	
ASR	−1.2	1.2		−5.8	5.8		−2.9	2.9	
**Age (y)**			93.72 (3) ^§^			29.20 (3) ^§^			5.28 (3)
≤ 25, *N* (%)	2,954 (97.7)	69 (2.3)		2,856 (94.5)	167 (5.5)		2,893 (95.7)	130 (4.3)	
ASR	5.8	−5.8		3.6	−3.6		−1.0	1.0	
26–35, *N* (%)	7,020 (96.6)	249 (3.4)		6,797 (93.5)	472 (6.5)		7,007 (96.4)	262 (3.6)	
ASR	4.4	−4.4		2.3	−2.3		2.1	−2.1	
36–45, *N* (%)	4,146 (95.0)	218 (5.0)		4,028 (92.3)	336 (7.7)		4,189 (96.0)	175 (4.0)	
ASR	−2.8	2.8		−2.0	2.0		−0.1	0.1	
>45, *N* (%)	3,615 (93.5)	250 (6.5)		3,537 (91.5)	328 (8.5)		3,695 (95.6)	170 (4.4)	
ASR	−7.7	7.7		−4.0	4.0		−1.5	1.5	
**Education**			7.60 (3)			9.88 (3) ^†^			4.44 (3)
College, *N* (%)	2,810 (95.7)	127 (4.3)		2,765 (94.1)	172 (5.9)		2,834 (96.5)	103 (3.5)	
ASR	−0.2	0.2		2.7	−2.7		1.4	−1.4	
Undergraduate, N (%)	13,587 (95.8)	591 (4.2)		13,163 (92.8)	1,015 (7.2)		13,602 (95.9)	576 (4.1)	
ASR	0.9	−0.9		−1.2	1.2		−1.0	1.0	
Master Degree, *N* (%)	1,281 (95.5)	61 (4.5)		1,232 (91.8)	110 (8.2)		1,289 (96.1)	53 (3.9)	
ASR	−0.6	0.6		−1.7	1.7		0.1	−0.1	
Ph.D., *N* (%)	57 (89.1)	7 (10.9)		58 (90.6)	6 (9.4)		59 (92.2)	5 (7.8)	
ASR	−2.7	2.7		−0.7	0.7		−1.6	1.6	
**School section**			38.83 (3) ^§^			27.72 (3) ^§^			37.45 (3) ^§^
Kindergarten, *N* (%)	1,388 (96.7)	48 (3.3)		1,360 (94.7)	76 (5.3)		1,376 (95.8)	60 (4.2)	
ASR	1.8	−1.8		2.7	−2.7		−0.4	0.4	
Primary school, *N* (%)	9,400 (96.3)	364 (3.7)		9,107 (93.3)	657 (6.7)		9,440 (96.7)	324 (3.3)	
ASR	3.7	−3.7		1.7	−1.7		4.9	−4.9	
Middle school, *N* (%)	6,651 (95.1)	343 (4.9)		6,466 (92.5)	528 (7.5)		6,669 (95.4)	325 (4.6)	
ASR	−3.5	3.5		−2.1	2.1		−3.6	3.6	
University, *N* (%)	296 (90.5)	31 (9.5)		285 (87.2)	42 (12.8)		299 (91.4)	28 (8.6)	
ASR	−4.7	4.7		−4.1	4.1		−4.3	4.3	

* *The scores of GAD-7 < 5, ≥ 5, ≥ 10, and ≥ 15 represent non, mild, moderate, and severe anxiety symptoms, respectively*.

** *The scores of PHQ-15 <5, ≥ 5, ≥ 10, and ≥ 15 represent non, mild, moderate, and severe somatic symptoms, respectively*.

*** *PHQ-2 score ≥ 3 indicates clinically significant depressive symptoms*.

**** *Adjusted Standardized Residuals. The larger the ASR, the larger the contribution of the cell to the overall chi-square test. We set ± 3 as a significant difference*.

† *p ≤ 0.05*,

‡ *p ≤ 0.01*,

§ *p ≤ 0.001*.

To analyze the correlation of suicidal thoughts and sleep disturbance with demographics, we separated subjects into two groups using symptom duration of half of the days as threshold (Table 3). Age is the only known factor associated with the severity of suicidal thoughts (X^2^ = 12.22, *P* < 0.01). Teachers younger than 25 and above 45 are more likely to have severe suicidal thoughts than those in between. For sleep disturbance, there were statistical differences among different genders, education backgrounds, and school sections. More women complained about serious sleep problem. So do university teachers and those with PhD degrees.

**Table 3:** Demographic and Sleep disturbance and Suicidal thoughts, Chi-square analysis.

	Sleep disturbance^*^		**χ^2^ (df)**	Suicidal thoughts^**^		**χ^2^ (df)**
**Demographic Characteristics**	**Non or less than half of the days *N =* 17,193**	**Over half of the days *N =* 1,328**		**Non or less than half of the days *N =* 18,422**	**Over half of the days *N =* 99**	
**Sex**			24.76 (3) ^§^			0.00 (1)
Men, *N* (%)	3,700 (94.7)	209 (5.3)		3,888 (99.5)	21 (0.5)	
ASR^***^	5.0	−5.0		0.0	0.0	
Women, *N* (%)	13,493 (92.3)	1,119 (7.7)		14,534 (99.5)	78 (0.5)	
ASR	−5.0	5.0		0.0	0.0	
**Age (y)**			2.74 (3)			12.22 (3) ^‡^
≤ 25, *N* (%)	2,797 (92.5)	226 (7.5)		3,003 (99.3)	20 (0.7)	
ASR	−0.7	0.7		−1.0	1.0	
26–35, *N* (%)	6,760 (93.0)	509 (7.0)		7,244 (99.7)	25 (0.3)	
ASR	0.7	−0.7		2.9	−2.9	
36–45, *N* (%)	4,066 (93.2)	298 (6.8)		4,342 (99.5)	22 (0.5)	
ASR	1.0	−1.0		0.3	−0.3	
>45, *N* (%)	3,570 (92.4)	295 (7.6)		3,833 (99.2)	32 (0.8)	
ASR	−1.3	1.3		−2.8	2.8	
**Education**			11.67 (3) ^‡^			3.39 (3)
College, *N* (%)	2,738 (93.2)	199 (6.8)		2,918 (99.4)	19 (0.6)	
ASR	0.9	−0.9		−0.9	0.9	
Undergraduate, *N* (%)	13,167 (92.9)	1,011 (7.1)		14,103 (99.5)	75 (0.5)	
ASR	0.4	−0.4		0.2	−0.2	
Master Degree, *N* (%)	1,235 (92.0)	107 (8.0)		1,338 (99.7)	4 (0.3)	
ASR	−1.2	1.2		1.2	−1.2	
Ph.D., *N* (%)	53 (82.8)	11 (17.2)		63 (98.4)	1 (1.6)	
ASR	−3.1	3.1		−1.1	1.1	
**School section**			36.80 (3) ^§^			2.02 (3)
Kindergarten, *N* (%)	1,349 (93.9)	87 (6.1)		1,431 (99.7)	5 (0.3)	
ASR	1.7	−1.7		1.0	−1.0	
Primary school, *N* (%)	9,049 (92.7)	715 (7.3)		9,715 (99.5)	49 (0.5)	
ASR	−0.8	0.8		0.6	−0.6	
Middle school, *N* (%)	6,518 (93.2)	476 (6.8)		6,951 (99.4)	43 (0.6)	
ASR	1.5	−1.5		−1.2	1.2	
University, *N* (%)	277 (84.7)	50 (15.3)		325 (99.4)	2 (0.6)	
ASR	−5.7	5.7		−0.2	0.2	

* *The sleep item asked the subjects how many days they had a problem of “difficulty falling asleep, difficulty sleeping, or excessive sleep” in the past 2 weeks*.

** *The sleep item asked the subjects how many days they had self-harm or suicidal thoughts in the past 2 weeks*.

*** *Adjusted Standardized Residuals. The larger the ASR, the larger the contribution of the cell to the overall chi-square test. We set ± 3 as a significant difference*.

† *p ≤ 0.05*,

‡ *p ≤ 0.01*,

§ *p ≤ 0.001*.

### Preference of Psychological Intervention

The most preferred psychological intervention is the practice of stress management skills (62.0%). 32.3% teachers would like to download and read psychology education materials, 5.1% preferred to use telephone hotline, and 19.1% thought they needed online psychological counseling. Table 4 shows the Chi-square analysis of symptomology and treatment preference. Individuals with non to mild symptoms seem to be satisfied with both psychology reading materials and stress management skills; those with moderate and severe symptoms are more likely in need of hotline and online counseling. For anxiety, somatic discomfort, depression and sleep disturbance, teachers with moderate to severe problems are more likely to choose an external intervention. For PTSD, 62% of asymptomatic and 63.5% of symptomatic teachers chose stress management skills but there was no difference between two groups. For suicidal thoughts, the only association found was for telephone hotline use. Teachers with serious suicidal thoughts are three times more likely to use telephone hotline (X^2^= 25.29, *P* ≤ 0.001). This difference was not detected for other types of interventions.

**Table 4:** The association between Treatment preference and GAD-7, PHQ-15, PHQ-2, SI, Sleep disturbance Chi-square analysis.

	Psychological materials^*^		**χ^2^ (df = 1)**	Stress management skills^**^		**χ^2^ (df = 1)**	**Telephone hotline**		**χ^2^ (df = 1)**	**Online psychological counseling**		**χ^2^ (df = 1)**
	**No need *N* = 12,545**	**Need *N =* 5,976**		**No need *N =* 7,044**	**Need *N =* 11,477**		**No need *N =* 17,579**	**Need *N =* 942**		**No need *N =* 14,989**	**Need *N =* 3,532**	
**Anxiety symptoms**			5.61^†^			51.89^§^			50.95^§^			20.76^§^
Non to mild, *N* (%)	12,043 (67.9)	5,692 (32.1)		6,841 (38.6)	10,894 (61.4)		16,876 (95.2)	859 (4.8)		14,402 (81.2)	3,333 (18.8)	
ASR^***^	2.4	−2.4		7.2	−7.2		7.1	−7.1		4.6	−4.6	
Moderate to severe, *N* (%)	502 (63.9)	284 (36.1)		203 (25.8)	583 (74.2)		703 (89.4)	83 (10.6)		587 (74.7)	199 (25.3)	
ASR	−2.4	2.4		−7.2	7.2		−7.1	7.1		−4.6	4.6	
**Somatization**			11.67^§^			77.31^§^			53.11^§^			38.21^§^
Non to mild, *N* (%)	11,718 (68.1)	5,500 (31.9)		6,697 (38.9)	10,521 (61.1)		16,398 (95.2)	820 (4.8)		14,019 (81.4)	3,199 (18.6)	
ASR	3.4	−3.4		8.8	−8.8		7.3	−7.3		6.2	−6.2	
Moderate to severe, *N* (%)	827 (63.5)	476 (36.5)		347 (26.6)	956 (73.4)		1,181 (90.6)	122 (9.4)		970 (74.4)	333 (25.6)	
ASR	−3.4	3.4		−8.8	8.8		−7.3	7.3		−6.2	6.2	
**PTSD**			10.82^§^			0.10			43.23^§^			4.02^†^
Asymptomatic, *N* (%)	12,495 (67.8)	5,930 (32.2)		7,009 (38.0)	11,416 (62.0)		17,502 (95.0)	923 (5.0)		14,919 (81.0)	3,506 (19.0)	
ASR	3.3	−3.3		0.3	−0.3		6.6	−6.6		2.0	−2.0	
Possible PTSD, *N* (%)	50 (52.1)	46 (47.9)		35 (36.5)	61 (63.5)		77 (80.2)	19 (19.8)		70 (72.9)	26 (27.1)	
ASR	−3.3	3.3		−0.3	0.3		−6.6	6.6		−2.0	2.0	
**Depressive symptoms**			10.98^§^			36.76^§^			58.01^§^			32.36^§^
Asymptomatic, *N* (%)	12,087 (68.0)	5,697 (32.0)		6,842 (38.5)	10,942 (61.5)		16,924 (95.2)	860 (4.8)		14,452 (81.3)	3,332 (18.7)	
ASR	3.3	−3.3		6.1	−6.1		7.6	−7.6		5.7	−5.7	
Symptomatic, *N* (%)	458 (62.1)	279 (37.9)		202 (27.4)	535 (72.6)		655 (88.9)	82 (11.1)		537 (72.9)	200 (27.1)	
ASR	−3.3	3.3		−6.1	6.1		−7.6	7.6		−5.7	5.7	
**Sleep disturbance**			10.23^§^			97.23^§^			57.42^§^			9.16^‡^
< half of the days, *N* (%)	11,698 (68.0)	5,495 (32.0)		6,707 (39.0)	10,486 (61.0)		16,377 (95.3)	816 (4.7)		13,956 (81.2)	3,237 (18.8)	
ASR	3.2	−3.2		9.9	−9.9		7.6	−7.6		3.0	−3.0	
≥half of the days, *N* (%)	847 (63.8)	481 (36.2)		337 (25.4)	991 (74.6)		1,202 (90.5)	126 (9.5)		1,033 (77.8)	295 (22.2)	
ASR	−3.2	3.2		−9.9	9.9		−7.6	7.6		−3.0	3.0	
**Suicidal thoughts**			1.19			0.30			25.29^§^			3.34
< half of the days, *N* (%)	12,483 (67.8)	5,939 (32.2)		7,009 (38.0)	11,413 (62.0)		17,496 (95.0)	926 (5.0)		14,916 (81.0)	3,506 (19.0)	
ASR	1.1	−1.1		0.6	−0.6		5.0	−5.0		1.8	−1.8	
≥half of the days, *N* (%)	62 (62.6)	37 (37.4)		35 (35.4)	64 (64.6)		83 (83.8)	16 (16.2)		73 (73.7)	26 (26.3)	
ASR	−1.1	1.1		−0.6	0.6		−5.0	5.0		−1.8	1.8	

* *Self-care and self-reading of psychological materials*.

** *Apply self-learned stress management skills*.

*** *Adjusted Standardized Residuals. The larger the ASR, the larger the contribution of the cell to the overall chi-square test. We set ± 3 as a significant difference*.

† *p ≤ 0.05*,

‡ *p ≤ 0.01*,

§ *p ≤ 0.001*.

Also, more male teachers chose self-care and self-reading of psychological materials (35.5% vs. 31.4%), telephone hotline (6.3% vs. 4.8%), and online counseling (20.3% vs. 18.8%). To the opposite, significantly more females (62.8% vs. 20.0%, *P* ≤ 0.001) preferred stress management skills. We also found that telephone hotline service and online counseling were preferred choices of teachers between 26 and 35 comparing with other age groups, and the former was also more liked by those who teach in the middle and high school settings.

## Discussion

The main purpose of this study is to examine the mental health impact of COVID-19 pandemic on Chinese teachers during school closure time. There are several findings in this study. First, of all teachers surveyed, more than 1/4 (25.3%) had mild or more somatic discomfort, 1/6 (17.7 %) had mild to severe anxiety, only 4.0% reported clinically significant depression. This ratio is lower than the 34% anxiety and severe anxiety and 8% severe depression reported by the survey of middle school teachers at the beginning of the Greek pandemic (14). As study has found that Chinese patients with depression are more likely to report feelings of fatigue and muscle aches instead of psychological symptoms (15, 16), under-report of depression in this population cannot be ruled out. Comparing with 9.3% occurrence rate of the general population (17), high level of anxiety may be related to the high infectivity of COVID-19 and the rapid information exchange under modern social media technology. During the SARS outbreak in 2003, some studies pointed out that the level of anxiety was closely related to the intensity of the outbreak and the number of new cases every day (18). This pandemic is the first major one in the social media age. Early and quick epidemiological analysis proved that the spread of 2019-nCoV is much faster than previous outbreaks of SARS-CoV and MERS-CoV (2). This and other coronavirus related information, both true and false, were quickly shared on the Internet and various social media platforms. Human takes cues and feedbacks from each other. The society's perception and response to a disaster like COVID-19 can be easily changed by publicized information. The unprecedented myriad real-time postings on social media, including the rapidly growing cases every day, can undoubtedly increase public anxiety and frustration. Additionally, from the beginning of this crisis, so-called draconian quarantine measures against the novel coronavirus limited people's normal social contact, which may also be closely related to the emergence of anxiety among the studied population (19).

Second, sleep disturbance, general fatigue and back pain were the three most frequently reported somatic symptoms in teachers. This is consistent with many previous somatization studies (17, 20, 21). The sleep complaint was also self-validated by a separate sleep item in the survey. Sleep is crucial to human life and closely related to emotion. Poor sleep is a risk factor for depression (22), anxiety (23), suicidal behavior (24), and PTSD symptoms (25). Long-term lack of sleep is also associated with fatigue and body aches, which in turn can lead to a decline in mental or physical functionality and be strong predictors of depression (26). Even though only 4.0% of the teachers interviewed had significant depressive symptoms, high prevalence of sleep problem and fatigue indicates more people could potentially escalate into clinical depression if untreated. Given the fact that both are important risk factors for suicidal thoughts and behavior, we can at least partially explain the reason why 2.8% of the respondents reported self-injury or suicidal thoughts. This study calls for a great attention to teachers' sleep needs and fatigue during COVID-19. At early stage, promoting good sleep hygiene and giving self-help tips to manage stress and fight overwhelming tiredness could be appropriate approach. Later on, clinical attention to the depression level of the teachers is certainly warranted.

Third, compared with 28.9% of PTSD symptoms among residents voluntarily quarantined in Toronto during SARS (27), and 24.55% of PTSD incidence among college teachers in Wuhan during COVID-19 (28), the rate was significantly lower in this survey. This may be related to the fact that this survey is done in Hunan, where the number of infections is relatively smaller than other major cities in China. Previous studies have shown that the occurrence of PTSD-related symptoms is related to the risk of virus exposure (29) and the fatality of the epidemic (30). The scientific data concerning the virus structure, transmission and epidemiology was quickly shared by the Chinese government after the outbreak. The lockdown of major cities like Wuhan and other mitigation measures helped people get better prepared, and may have increased their sense of control. All above plus early survey time could be the reasons for the low PTSD rate from this survey.

Fourth, the survey found that the female teachers reported more sleep disturbance, depression or somatic discomfort. This is consistent with the results of previous studies (18, 31–33). Research found that the periodic changes of estradiol and progesterone in women may make them more prone to some emotional problems (31, 34). Teachers over 45 years old are more likely to have moderate to severe somatic discomfort and anxiety, which may be related to the initial epidemic report that the middle-aged and elderly are more vulnerable to the coronavirus. The result again alerted these two groups worthy of more mental health attention.

The study also found teachers with higher degrees and university teachers are most vulnerable to various psychological symptoms. This may be related to the professional characteristics of these two groups. Previous studies have shown that Chinese university teachers are generally enduring higher level of stress due to research requirement, pressure of promotion, and lack of adequate rest. Long-term stress is associated with poor sleep and can also reduce an individual's sense of self-efficacy (35). It can cause individuals to be more susceptible to negative environmental impact, make them more likely to have negative subjective experience during the COVID-19 pandemic.

In China, the interest in seeking mental health help is largely hindered by many factors including strict social norms, cultural beliefs, stigma, mental health literacy, etc. (36–38). People with higher level of education are more likely to seek professional help (39, 40). We hypothesized that our study population, with higher education than average citizens, may have better attitudes toward mental health help. With an overarching aim to build an effective psychological support system for teachers during and after the pandemic, we listed four commonly used and practically implementable interventions for the participants to choose. Multiple selections were allowed. We found men teachers are more likely to choose psychological material reading and online counseling, and women teachers are more likely to choose practicing stress management skills. Teachers aged 26–35 prefer telephone hotlines and online counseling. This age group did not report higher rate of severe mental health symptoms. Their desire to seek telephone or online help may be associated with their acceptance of and familiarity with high technology. Middle/high school teachers are more likely to choose psychological materials and hotline service. Even if the underlying reason is unknown, we should consider providing more related education to middle/high schools during and past the pandemic.

We are not surprised to see that teachers with moderate to severe mental problems are more likely to seek help. Perceived high level of psychological distress may increase treatment seeking behavior. Even though majority of teachers tend not to choose telephone hotline as a way for help, those with serious self-harm and suicidal thoughts are more willing to use this method. This is encouraging because only through hotline can emergent help be achieved. Previous studies have shown that online screening may enhance decisions to seek professional help (41). We hope that this large-scale survey will increase public awareness of mental health concerns in teachers. A follow-up data showed that by the time of this manuscript, the psychological materials we provided during and after this survey has reached 341,539 electronic downloads, 5,155 online audios listening and 4,003 video views. Our hotline has received 762 calls between January 31 and April 6. This is a clear manifestation of mental health demand during the COVID-19 pandemic. We've learned that many teachers suffer from psychological symptoms that warrant different levels of intervention, and majority indicated that they needed more than one way of psychological assistance.

This study has strengths and limitations. First, this is a large-scale survey with a very high response. Second, we used several standardized instruments to investigate multiple aspects of teacher's mental health condition. Third, this is a timely research on a special population during COVID-19 pandemic. At the time of this manuscript, COVID-19 transmission has been better controlled but continues to be a global health emergency. Businesses and schools are reopening in many places. This study can be used as a reference in public mental health strategic planning and rapid deployment of effective mental health interventions. Limitations include, first, it is a cross sectional study so limited to a single time point. We were not able to do a pre- and-post COVID-19 comparison of the psychological distress of teachers. Second, this survey is targeted to teachers who generally have high level of education. The teaching requirements, expectations from students and parents, modal of remote education can vary from place to place. Therefore, many confounding factors existed so the study result may not be generalizable to a different population in other countries during the COVID-19 outbreak. Third, considering that the length of the questionnaire may affect the respondent's compliance with the questionnaire, we only use a single item to ask about sleep disorders. This item was previously used to screen for sleep problems in cancer patients, but it lacks the reliability test of the teacher population.

## Conclusions

This study investigated the mental health condition of Chinese teachers during the COVID-19 outbreak. The survey found that the major reported psychological distresses are anxiety, sleep disturbance, and somatic symptoms. Small number of teachers reported depression and post-traumatic stress. Some had thoughts of self-injury or suicide. There were gender, age and school setting differences. Females over 45 years old and university teachers were more vulnerable to various mental problems. Different individuals have different preferences for intervention methods, mostly based on the type and severity of their symptoms.

## Data Availability Statement

The raw data supporting the conclusions of this article will be made available by the authors, without undue reservation.

## Ethics Statement

The studies involving human participants were reviewed and approved by the Ethics Committee of the Second Xiangya Hospital of Central South University. The patients/participants provided their written informed consent to participate in this study.

## Author Contributions

XL and CP: data collection, literature review, and manuscript drafting. WX and LW: managed the ethical review process. ZW, XS, LL, and ZL: manuscript drafting and revision. All authors read and approved the final manuscript.

## Conflict of Interest

The authors declare that the research was conducted in the absence of any commercial or financial relationships that could be construed as a potential conflict of interest.
